# Association between matrix metalloproteinases polymorphisms and ovarian cancer risk: A meta-analysis and systematic review

**DOI:** 10.1371/journal.pone.0185456

**Published:** 2017-09-28

**Authors:** Xu-Ming Zhu, Wei-Feng Sun

**Affiliations:** Department of Medical Laboratory, Wuxi People’s Hospital affiliated with Nanjing Medical University, Wuxi, Jiangsu Province, China; Florida State University, UNITED STATES

## Abstract

**Background:**

Published data on the relationship between matrix metalloproteinases (MMPs) polymorphisms and ovarian cancer risk have implicated inconclusive results. To evaluate the role of MMPs polymorphisms in ovarian cancer risk, a meta-analysis and systematic review were performed.

**Methods:**

MMPs polymorphisms which could be quantitatively synthesized were involved in meta-analysis. Five comparison models (homozygote model, heterozygote model, dominant model, recessive model, additive model) were carried out, a subgroup analysis was performed to clarify heterogeneity source. The remaining polymorphisms which could not be quantitatively synthesized were involved in systematic review.

**Results:**

10 articles with 20 studies were included in this paper. Among those studies, 8 studies involving MMP1 rs1799750 and MMP3 rs34093618 could be meta-analyzed and 12 studies involving 12 polymorphisms could not. Meta-analysis showed that no associations were found between MMP1 rs1799750 (homozygote model: OR = 0.93, 95%CI = 0.70–1.23, P_OR_ = 0.60; heterozygote model: OR = 1.09, 95%CI = 0.78–1.54, P_OR_ = 0.61; dominant model: OR = 1.02, 95%CI = 0.83–1.25, P_OR_ = 0.84; recessive model: OR = 0.95, 95%CI = 0.75–1.21, P_OR_ = 0.67; additive model: OR = 1.00, 95%CI = 0.85–1.17, P_OR_ = 0.99), MMP3 rs34093618 (homozygote model: OR = 1.25, 95%CI = 0.70–2.24, P_OR_ = 0.46; heterozygote model: OR = 1.08, 95%CI = 0.51–2.31, P_OR_ = 0.84; dominant model: OR = 0.97, 95%CI = 0.68–1.38, P_OR_ = 0.85; recessive model: OR = 1.12, 95%CI = 0.69–1.80, P_OR_ = 0.65; additive model: OR = 1.01, 95%CI = 0.79–1.31, P_OR_ = 0.91) and ovarian cancer. Furthermore, similar results were detected in subgroup analysis. The systematic review on 12 polymorphisms suggested that MMP2 C-735T, MMP7 A-181G, MMP8 rs11225395, MMP9 rs6094237, MMP12 rs2276109, MMP20 rs2292730, MMP20 rs12278250, MMP20 rs9787933 might have a potential effect on ovarian cancer risk.

**Conclusions:**

In summary, polymorphisms of MMPs might not be associated with ovarian cancer risk. However, it is necessary to conduct more larger-scale, multicenter, and high-quality studies in the future.

## Introduction

Ovarian cancer is main cause of death with gynecological tumors worldwide, and is often at an advanced stage by the time of diagnosis and has metastasized throughout the peritoneal cavity [1–2]. In 2013, there were an estimated 22,240 new cases and 14,030 new deaths [3]. Despite continuous advances in ovarian cancer research, diagnosis, and clinical treatment during the past 30 years [4], it has been still hard to find a cost-effective screening strategy to significantly increase the survival rate for early-stage ovarian cancer.

Genome-wide association studies (GWAS) concerning genetic aetiology of cancer have established more than 150 regions associated with various specific cancers, which expand the current understanding of carcinogenesis mechanisms [5]. Alterations in genetic sequence, such as single-nucleotide substitutions, lead to cancer formation by biologically regulating a handful of molecular activities [6].

Matrix metalloproteinases (MMPs), a family of more than 20 zinc-dependent enzymes known to degrade extracellular matrix and basement membrane components [7], are not only a prerequisite for multiple steps of cancer development but also play important roles in cancer invasion and metastasis [8]. MMPs are correlated with ovarian cancer, with the levels of MMP-2, MMP-7 and MMP-9 elevated in ovarian cancer patients [9–10]. At genetic level, a number of studies have been carried out to assess the association between polymorphisms of MMPs and ovarian cancer risk [11–27], but the conclusions have been still conflicted and even contradictory. For example, study by Ju [19] showed no associations existed between MMP1 rs1799750 and ovarian cancer in Korean, while study by Kanamori [11] showed 2G genotype of MMP1 rs1799750 might represent a risk factor for ovarian cancer in Japanese. Individual studies with a small sample size may result in incorrect conclusion. Therefore, a comprehensive meta-analysis and systematic review are necessary to precisely assess the relationships between MMPs polymorphisms and ovarian cancer risk.

## Materials and methods

### Search strategy

The databases Pubmed, Embase, Web of knowledge, were searched for all articles with the following search terms: (MMP OR MMPs OR matrix metalloproteinase OR matrix metalloproteinases) AND (polymorphism OR polymorphisms) AND (ovarian cancer OR ovarian carcinoma) up to search date: March 25, 2017. No limitation of publication language was defined for this search. Additional published data were identified by reviewing the bibliographical references listed in each retrieved article.

### Inclusion criteria and exclusion criteria

All studies included in this meta-analysis were accorded with the following inclusion criteria: (a) study focused on the association between MMPs polymorphisms and ovarian cancer; (b) case-control design; (c) provided available frequency for each genotype in both cases and controls to calculate odds ratio (OR) and corresponding 95% confidence interval (95%CI). In addition, exclusion criteria were as follows: (a) reviews, editorials, comments or animal studies; (b) overlapped articles or studies with overlapping data.

### Data extraction

Two investigators independently extracted the following data: first author’s name, year of publication, study country, ethnicity, source of controls, MMPs gene, polymorphisms, number of cases and controls, value of Hardy-Weinberg equilibrium (HWE). A consensus on the extracted items was reached by discussion between the two investigators.

### Quality assessment

The quality of study was assessed according to the quality assessment criteria [28] (S1 Table), in which the quality scores ranged from 0 to 15. Studies with scores ≥9 were regarded as high quality.

### Statistical analysis

In order to evaluate the association between MMPs polymorphisms and ovarian cancer risk, OR and 95% CI were summarized under five comparison models, including homozygote model, heterozygote model, dominant model, recessive model, additive model. The definition of comparison model was listed in S2 Table. The P value of the pooled ORs was considered significant if less than 0.05, which was examined by Z test. HWE in the control group was checked by chi-square test, deviation was considered with P<0.05. Heterogeneity assumption was checked by a chi-square-based Q statistic test and quantified by I^2^ value. If I^2^ value < 50% or *P >* 0.10, the fixed effect model was used [29]. Otherwise, random effect model was carried out [30], then a subgroup analysis by ethnicity was performed. Both funnel plot and Egger’s test were performed to test whether publication bias existed or not, bias was considered with P<0.05 in Egger’s test. The statistical analyses for the present study were completed by Review Manager software 5.1 (the Nordic Cochrane Center, Rigshospitalet, Copenhagen, Denmark) and Stata software 12.0 (StataCorp, College Station, TX, USA).

## Results

### Literature search and study characteristics

**Fig 1:**
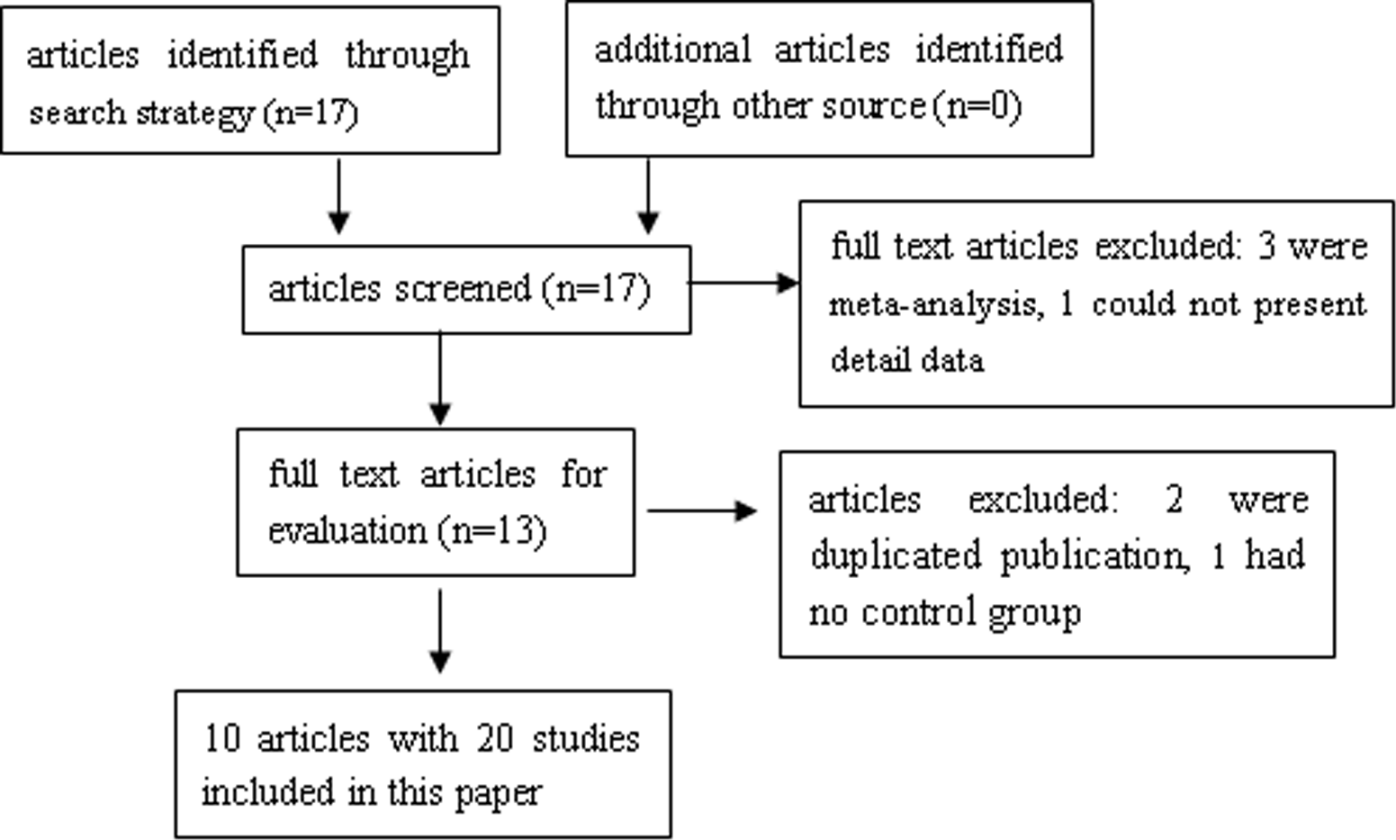
Flow diagram of study selection process.

A total of 17 articles [11–27] were identified through search strategy. Reviewed on abstracts among these articles, 4 articles were excluded because 3 articles [25–27] were meta-analysis and 1 article [13] could not present detailed data. Then 13 full text articles were obtained for further evaluation, in which 3 articles were deleted for 2 articles [16, 21] were duplicated publication and 1 article [17] had no control group. Ultimately, 10 articles with 20 studies involving 14 polymorphisms were included in this paper. Among these studies, 8 studies with 2 polymorphisms [11, 12, 14, 15, 18, 19] (5 studies for MMP1 rs1799750, 3 studies for MMP3 rs34093618) involving 1019 ovarian cancer cases and 1609 controls could be quantitatively synthesized for meta-analysis. The remaining 12 studies with 12 polymorphisms [18, 20, 22, 23, 24] (12 polymorphisms including MMP2 C-1306T, MMP2 C-735T, MMP7 A-181G, MMP8 rs2155052, MMP8 rs11225395, MMP9 C-1562T, MMP9 rs6094237, MMP12 rs2276109, MMP13 rs17860523, MMP20 rs2292730, MMP20 rs12278250, MMP20 rs9787933) involving 2793 ovarian cancer cases and 3037 controls could not be quantitatively synthesized, thus the systematic review was performed. The flow diagram of study selection process was presented in Fig 1. The main characteristics of included articles or studies were listed in Table 1. The distributions of genotype in studies from meta-analysis and systematic review were in S3 Table and S4 Table.

**Table 1:** Characteristics of studies included in the meta-analysis and systematic review.

first author	year	contry	ethnicity	source of control	gene	polymorphisms	sample sizes (case/control)	HWE	quality score
Kanamori [11]	1999	Japan	East Asia	NA	MMP1	rs1799750	163/150	0.009	5
Biondi [12]	2000	Italy	Caucasian	NA	MMP1	rs1799750	25/164	0.52	4
					MMP3	rs34093618	25/164	0.217	
Wenham [14]	2003	USA	mixed	PB	MMP1	rs1799750	311/387	0.264	12
Smolarz [15]	2003	Poland	Caucasian	HB	MMP3	rs34093618	118/110	0.587	8
Li [18]	2006	China	East Asia	HB	MMP1	rs1799750	122/151	0.002	9
					MMP3	rs34093618	122/151	0.275	
					MMP7	A-181G	138/160	0.714	
					MMP9	C-1562T	138/160	0.263	
Ju [19]	2007	Korea	East Asia	HB	MMP1	rs1799750	133/332	0.393	7
Li [20]	2008	China	East Asia	PB	MMP2	C-1306T	246/324	0.862	10
					MMP2	C-735T	246/324	0.293	
Jia [22]	2010	China	East Asia	HB	MMP12	rs2276109	300/300	0.746	12
					MMP13	rs17860523	300/300	0.962	
Arechavaleta-Velasco [23]	2014	Mexico	mixed	NA	MMP8	rs2155052	35/37	0.797	6
					MMP8	rs11225395	35/37	0.013	
Wang [24]	2015	USA	mixed	HB	MMP9	rs6094237	339/349	0.049	12
					MMP20	rs2292730	339/349	0.01	
					MMP20	rs12278250	339/349	0.675	
					MMP20	rs9787933	339/349	0.59	

NA, not available; HB, hospital based; PB, population based; MMP, matrix metalloproteinase; HWE, Hardy-Weinberg equilibrium

### Meta-analysis and systematic review

**Fig 2:**
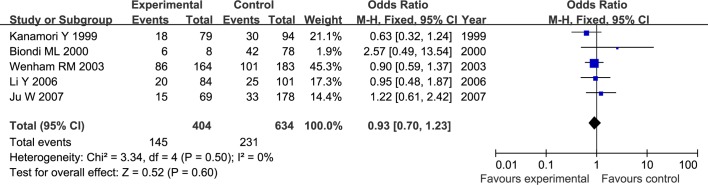
Forest plot of MMP-1 rs1799750 and ovarian cancer risk (1G1G vs 2G2G).

**Fig 3:**
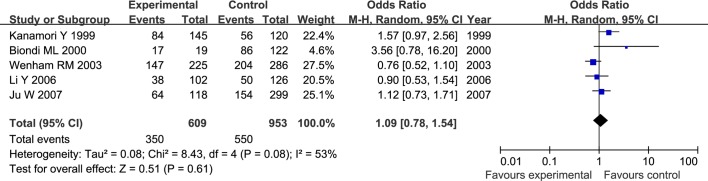
Forest plot of MMP-1 rs1799750 and ovarian cancer risk (1G2G vs 2G2G).

**Fig 4:**
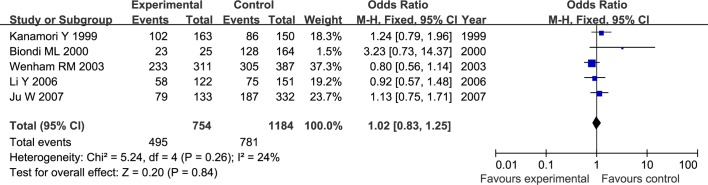
Forest plot of MMP-1 rs1799750 and ovarian cancer risk (1G1G +1G2G vs 2G2G).

**Fig 5:**
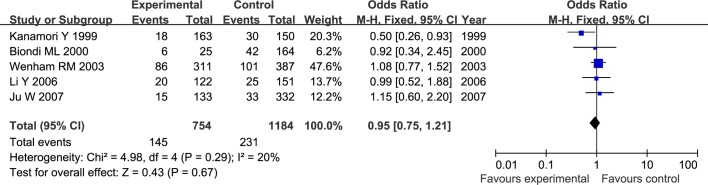
Forest plot of MMP-1 rs1799750 and ovarian cancer risk (1G1G vs 1G2G+2G2G).

**Fig 6:**
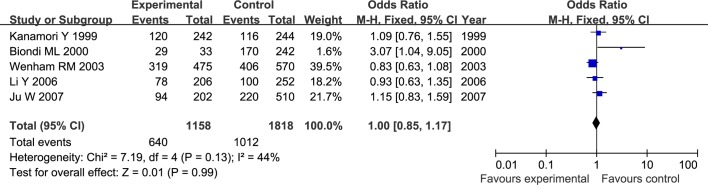
Forest plot of MMP-1 rs1799750 and ovarian cancer risk (1G vs 2G).

**Fig 7:**
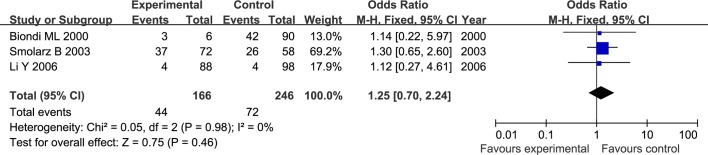
Forest plot of MMP3 rs34093618 and ovarian cancer risk (5A5A vs 6A6A).

**Fig 8:**
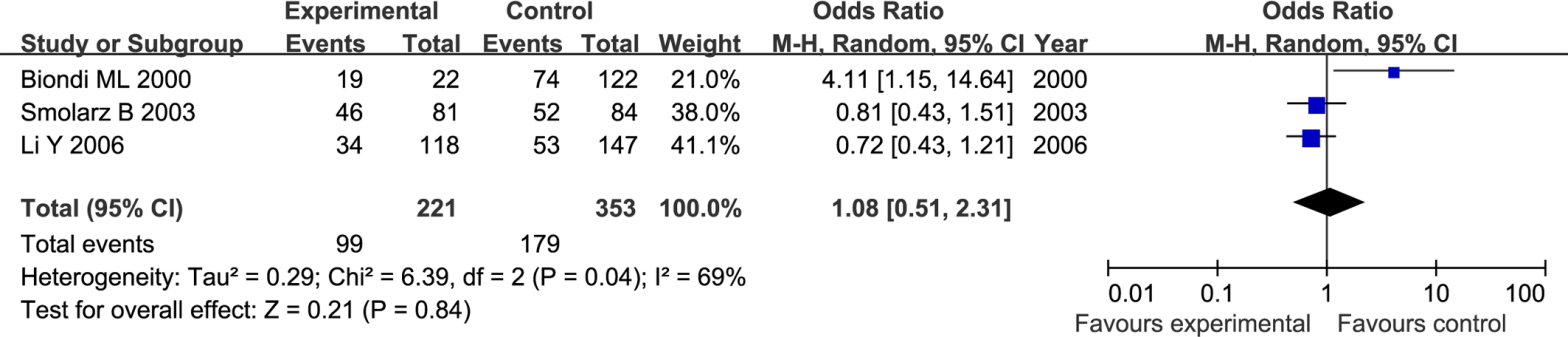
Forest plot of MMP3 rs34093618 and ovarian cancer risk (5A6A vs 6A6A).

**Fig 9:**
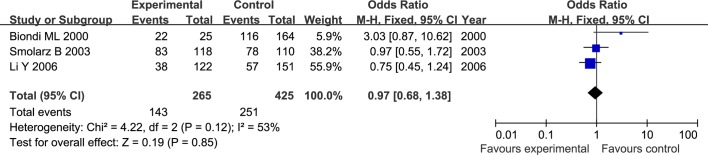
Forest plot of MMP3 rs34093618 and ovarian cancer risk (5A5A+5A6A vs 6A6A).

**Fig 10:**
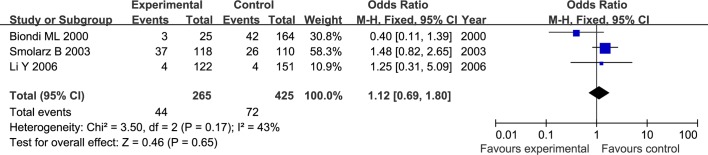
Forest plot of MMP3 rs34093618 and ovarian cancer risk (5A5A vs 5A6A + 6A6A).

**Fig 11:**
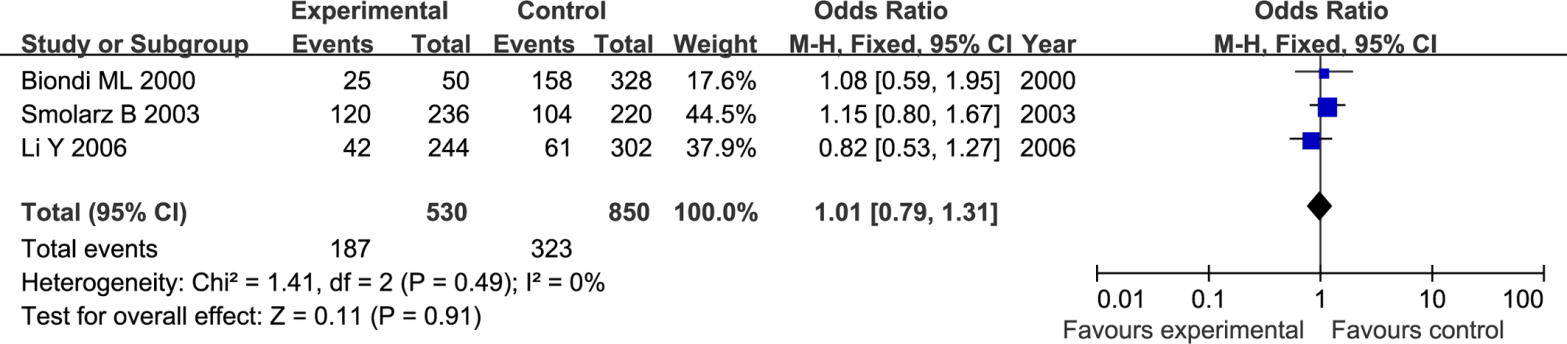
Forest plot of MMP3 rs34093618 and ovarian cancer risk (5A vs 6A).

The results of meta-analysis for MMP1 rs1799750 and MMP3 rs34093618 polymorphisms were listed in Table 2. The forest plots for MMP1 rs1799750 were listed in Figs 2–6, and MMP3 rs34093618 were presented in Figs 7–11. On the whole, no significant association was found between MMP1 rs1799750 polymorphisms and ovarian cancer risk (homozygote model: OR = 0.93, 95%CI = 0.70–1.23, P_OR_ = 0.60; heterozygote model: OR = 1.09, 95%CI = 0.78–1.54, P_OR_ = 0.61; dominant model: OR = 1.02, 95%CI = 0.83–1.25, P_OR_ = 0.84; recessive model: OR = 0.95, 95%CI = 0.75–1.21, P_OR_ = 0.67; additive model: OR = 1.00, 95%CI = 0.85–1.17, P_OR_ = 0.99). For MMP3 rs34093618 polymorphism and ovarian cancer risk, overall, no significant association was found (homozygote model: OR = 1.25, 95%CI = 0.70–2.24, P_OR_ = 0.46; heterozygote model: OR = 1.08, 95%CI = 0.51–2.31, P_OR_ = 0.84; dominant model: OR = 0.97, 95%CI = 0.68–1.38, P_OR_ = 0.85; recessive model: OR = 1.12, 95%CI = 0.69–1.80, P_OR_ = 0.65; additive model: OR = 1.01, 95%CI = 0.79–1.31, P_OR_ = 0.91).

**Table 2:** Meta-analysis of association between MMPs polymorphism and ovarian cancer.

comparison model	OR(95%CI)	P_OR_^a^	I^2^	P_het_^b^
MMP1 rs1799750				
1G1G vs 2G2G	0.93(0.70–1.23)	0.60	0%	0.50
1G2G vs 2G2G	1.09(0.78–1.54)	0.61	53%	0.08
1G1G+1G2G vs 2G2G	1.02(0.83–1.25)	0.84	24%	0.26
1G1G vs 1G2G+2G2G	0.95(0.75–1.21)	0.67	20%	0.29
1G vs 2G	1.00(0.85–1.17)	0.99	44%	0.13
MMP3 rs34093618				
5A5A vs 6A6A	1.25(0.70–2.24)	0.46	0	0.98
5A6A vs 6A6A	1.08(0.51–2.31)	0.84	69	0.04
5A5A+5A6A vs 6A6A	0.97(0.68–1.38)	0.85	53	0.12
5A5A vs 5A6A+6A6A	1.12(0.69–1.80)	0.65	43	0.17
5A vs 6A	1.01(0.79–1.31)	0.91	0	0.49

^a^ P value of the Z-test for odds ratio test

^b^ P value of the Q-test for heterogeneity test.

The results of systematic review were presented in Table 3. Eight polymorphisms (MMP2 C-735T, MMP7 A-181G, MMP8 rs11225395, MMP9 rs6094237, MMP12 rs2276109, MMP20 rs2292730, MMP20 rs12278250, MMP20 rs9787933) were reported associated with ovarian cancer risk, while other polymorphisms could not be associated with ovarian cancer risk.

**Table 3:** Systematic review of association between MMPs polymorphisms and ovarian cancer.

A										
gene	polymorphisms	homozygote model			heterozygote model			dominant model		
		OR(95%CI)	P_OR_^a^		OR(95%CI)		P_OR_^a^	OR(95%CI)		P_OR_^a^
MMP7	A-181G	NA	NA		NA		NA	NA		NA
MMP9	C-1562T	0.16(0.01, 3.46)	0.25		0.20(0.01, 4.37)		0.31	0.17(0.01, 3.57)		0.25
MMP2	C-1306T	3.86(0.45, 33.29)	0.22		3.78(0.43, 33.3)		0.23	3.84(0.45, 33.08)		0.22
MMP2	C-735T	1.12(0.52, 2.39)	0.78		0.67(0.30, 1.47)		0.32	0.93(0.44, 1.97)		0.85
MMP12	rs2276109	NA	NA		NA		NA	NA		NA
MMP13	rs17860523	0.64(0.41, 1.02)	0.06		0.84(0.56, 1.26)		0.40	0.77(0.52, 1.12)		0.17
MMP8	rs2155052	NA	NA		NA		NA	NA		NA
MMP8	rs11225395	0.38(0.08, 1.78)	0.22		0.24(0.07, 0.79)		0.02	0.26(0.08, 0.85)		0.03
MMP9	rs6094237	2.00(1.28, 3.12)	0.002		1.82(1.18, 2.980)		0.007	1.90(1.27, 2.85)		0.002
MMP20	rs2292730	0.53(0.34, 0.83)	0.005		0.47(0.32, 0.70)		0.0002	0.49(0.34, 0.72)		0.0002
MMP20	rs12278250	0.81(0.18, 3.66)	0.79		0.38(0.08, 1.77)		0.22	0.73(0.16, 3.28)		0.68
MMP20	rs9787933	1.46(0.32, 6.57)	0.62		0.72(0.15, 3.37)		0.68	1.29(0.29, 5.83)		0.74
B										
gene	polymorphisms	recessive model			additive model					
		OR(95%CI)	P_OR_^a^		OR(95%CI)		P_OR_^a^			
MMP7	A-181G	0.28(0.13, 0.63)	0.002		0.30(0.14, 0.67)		0.003			
MMP9	C-1562T	0.76(0.42, 1.38)	0.37		0.73(0.42, 1.26)		0.25			
MMP2	C-1306T	1.07(0.72, 1.58)	0.74		1.11(0.78, 1.59)		0.55			
MMP2	C-735T	1.58(1.12, 2.23)	0.009		1.36(1.02, 1.81)		0.04			
MMP12	rs2276109	0.36(0.17, 0.73)	0.005		0.37(0.18, 0.74)		0.005			
MMP13	rs17860523	0.72(0.50, 1.04)	0.08		0.80(0.61, 1.01)		0.06			
MMP8	rs2155052	1.46(0.23, 9.28)	0.69		1.94(0.34, 10.96)		0.45			
MMP8	rs11225395	1.07(0.31, 3.69)	0.92		0.63(0.33, 1.22)		0.17			
MMP9	rs6094237	1.30(0.95, 1.77)	0.10		1.37(1.10, 1.70)		0.004			
MMP20	rs2292730	0.91(0.65, 1.28)	0.60		0.76(0.62, 0.94)		0.01			
MMP20	rs12278250	2.02(1.31, 3.11)	0.001		1.79(1.20, 2.67)		0.004			
MMP20	rs9787933	1.99(1.33, 2.97)	0.0008		1.83(1.26, 2.66)		0.002			

NA, not available

^a^ P value of the Z-test for odds ratio test

### Heterogeneity analysis and subgroup analysis

**Fig 12:**
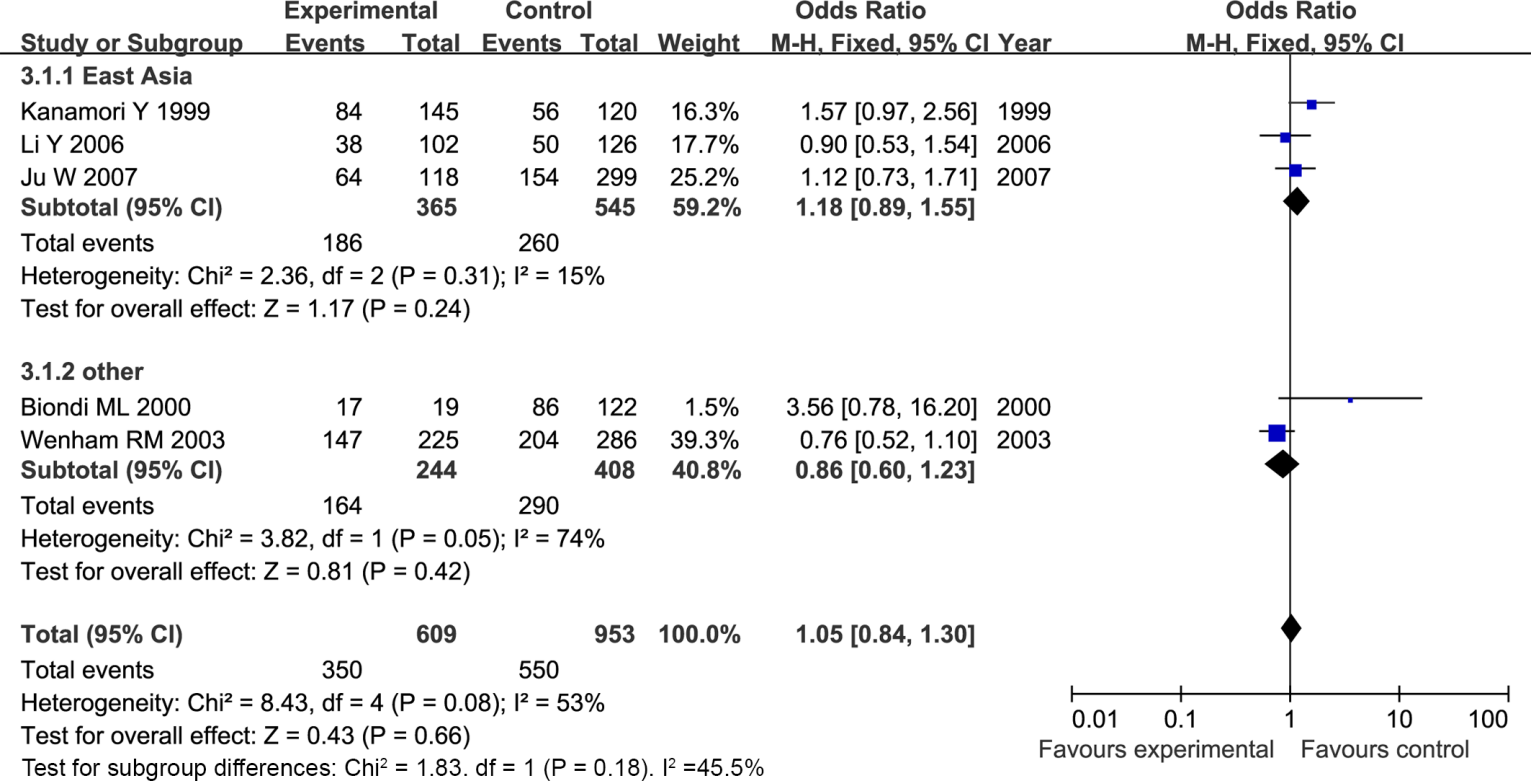
Forest plot of MMP-1 rs1799750 and ovarian cancer risk stratified according to ethnicity (1G2G vs 2G2G).

**Fig 13:**
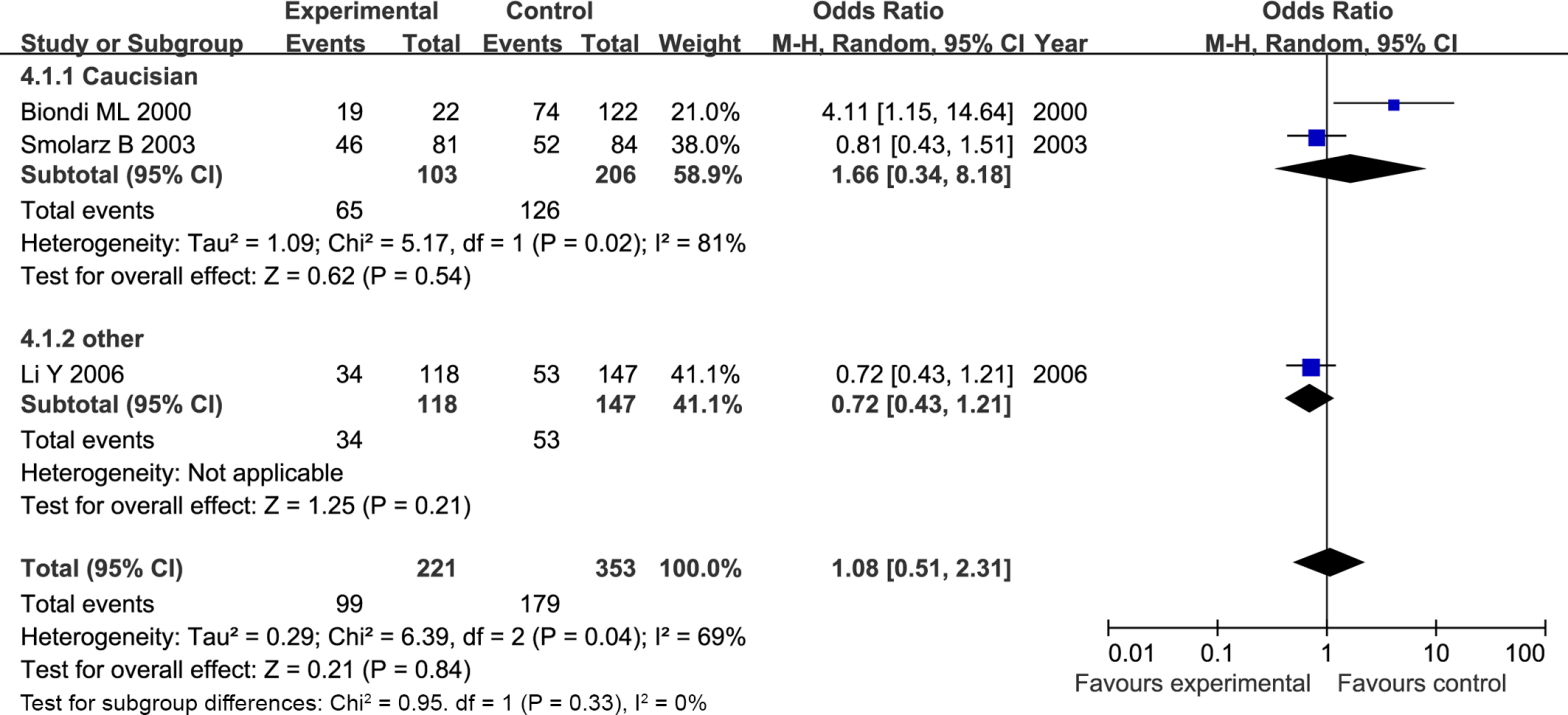
Forest plot of MMP3 rs34093618 and ovarian cancer risk stratified according to ethnicity (5A6A vs 6A6A).

For both MMP1 rs1799750 and MMP3 rs34093618 polymorphism, there was obvious heterogeneity in heterozygote model (MMP1 rs1799750: I^2^ = 53%, P_het_ = 0.08; MMP3 rs34093618: I^2^ = 69%, P_het_ = 0.04). Then, a subgroup analysis by ethnicity was conducted to assess the source of heterogeneity. The forest plots of subgroup analysis for MMP1 rs1799750 and MMP3 rs34093618 were respectively presented in Figs 12 and 13. For MMP1 rs1799750, heterogeneity dramatically decreased when stratification analyses for Caucasian was conducted (I^2^ = 31%, P_het_ = 0.15), while MMP3 rs34093618 did not decreased (I^2^ = 81%, P_het_ = 0.02). No significant association was found between MMPs polymorphism and ovarian cancer in both two subgroup analysis.

### Publication bias analysis

**Fig 14:**
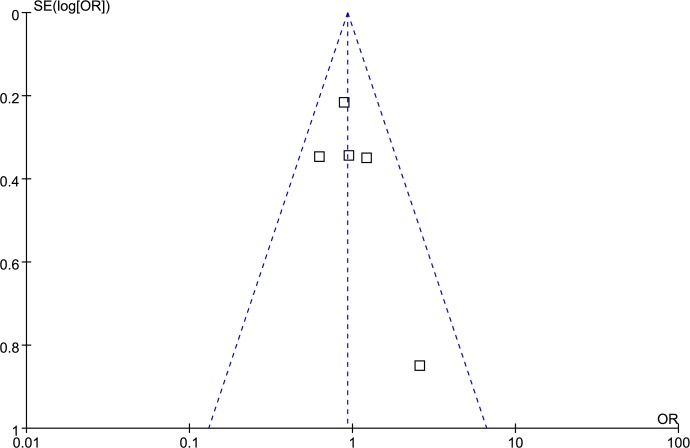
Funnel plot of MMP-1 rs1799750 and ovarian cancer risk (1G1G vs 2G2G).

**Fig 15:**
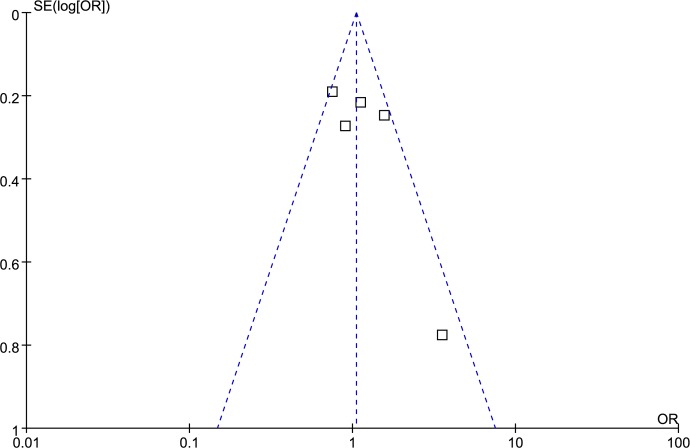
Funnel plot of MMP-1 rs1799750 and ovarian cancer risk (1G2G vs 2G2G).

**Fig 16:**
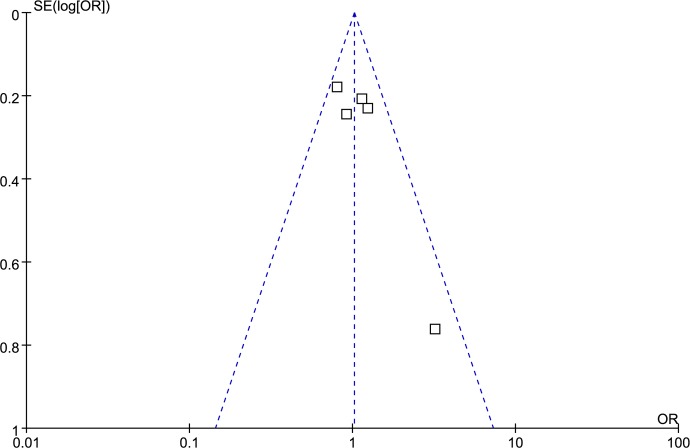
Funnel plot of MMP-1 rs1799750 and ovarian cancer risk (1G1G +1G2G vs 2G2G).

**Fig 17:**
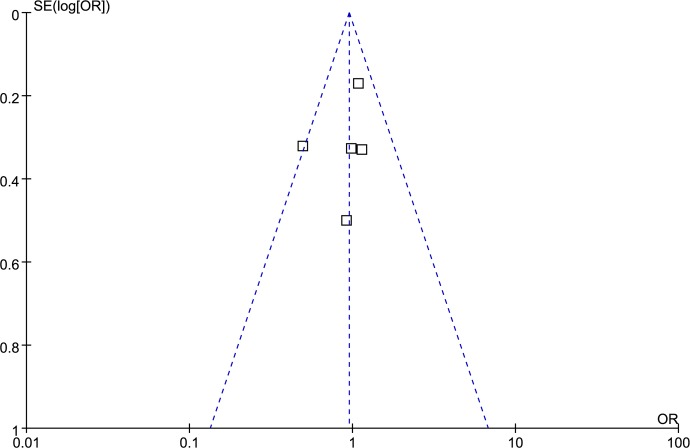
Funnel plot of MMP-1 rs1799750 and ovarian cancer risk (1G1G vs 1G2G+2G2G).

**Fig 18:**
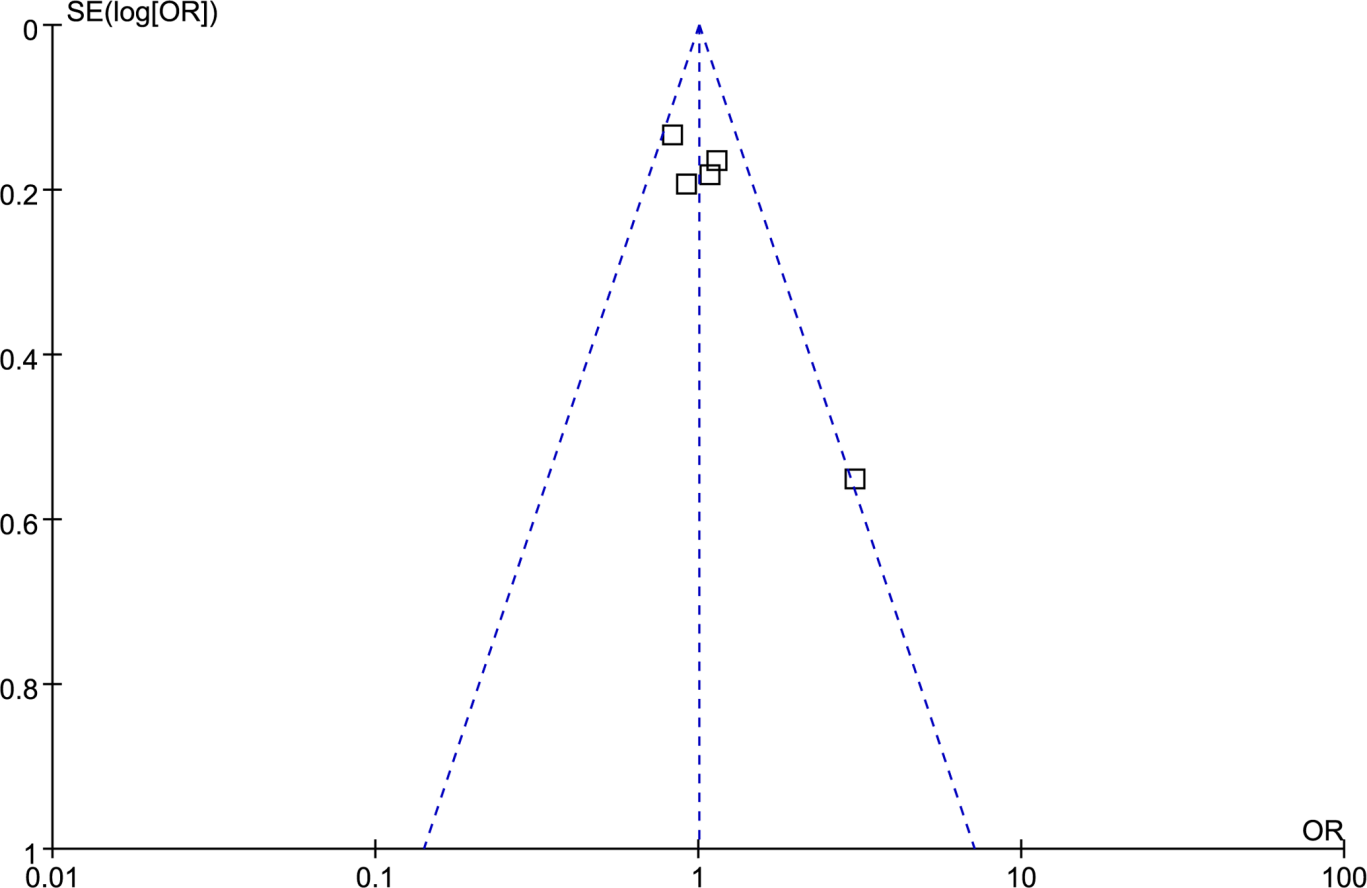
Funnel plot of MMP-1 rs1799750 and ovarian cancer risk (1G vs 2G).

Funnel plot and Egger’s test were performed to access publication bias. Both funnel plots (Figs 14–18) and Egger’s test (homozygote model: P = 0.588; heterozygote model: P = 0.423; dominant model: P = 0.612; recessive model: P = 0.363; additive model: P = 0.534) suggested no evidence of publication bias in the meta-analysis of MMP1 rs1799750 polymorphism. For MMP3 rs34093618, publication bias analysis was not conducted for only 3 studies involved.

## Discussion

Study by Ju [19] showed no associations existed between MMP1 rs1799750 and ovarian cancer in Korean, while study by Kanamori [11] showed 2G genotype of MMP1 rs1799750 might represent a risk factor for ovarian cancer in Japanese. Therefore, a comprehensive meta-analysis and systematic review are necessary. As a powerful tool for summarizing the different studies, meta-analysis has been accepted as a significant tool to analyze cumulative data from limited study subjects [31].

This meta-analysis and systematic review, including 5 studies for MMP1 rs1799750 composed of 754 ovarian cancer cases 1184 and controls, 3 studies for MMP3 rs34093618 polymorphism composed of 265 cases and 425 controls, 12 studies for systematic review involving 2793 cases and 3037 controls, proved that MMP1 rs1799750 and MMP3 rs34093618 polymorphisms were not associated with ovarian cancer risk, in addition, subgroup analyses by ethnicity showed similar results. Although in systematic review eight polymorphisms, including MMP2 C-735T, MMP7 A-181G, MMP8 rs11225395, MMP9 rs6094237, MMP12 rs2276109, MMP20 rs2292730, MMP20 rs12278250, MMP20 rs9787933, might be associated with ovarian cancer risk, it was inconclusive results due to lack of relevant studies. Except eight above polymorphisms, it was revealed that other four polymorphisms in systematic review were not related with ovarian cancer risk.

The major strengths of our study were its comprehensive and systematic focus on the relationship between MMPs polymorphisms and ovarian cancer risk. Although a meta-analysis by Wang [32] has also investigated the relationship of MMP1 rs1799750 polymorphism with ovarian cancer (5 studies involving 754 cases and 1184 control) and produced similar results, our report identified 15 additional studies including 3058 cases and 3462 controls, which have not been included in report of Wang [32].

Also, some limitations still existed in our paper. First, control group was not uniformly defined, some controls were population-based while other controls were hospital-based. Second, significant heterogeneity was observed in a few comparison models. Although a subgroup analysis was performed to clarify sources, it was hard to find all potential sources. Third, departure from HWE was detected in some studies. Finally, there was a lack of a unified criterion for including studies, leading to failure to adjust them in age and lifestyle et al.

In summary, our reports showed that MMPs polymorphisms might not be associated with ovarian cancer risk. However, it is necessary to conduct more larger-scale, multicenter, and high-quality studies in the future.

## Supporting information

S1 Table Score of quality assessment. (DOCX)

S2 Table Definition of comparison model. (DOCX)

S3 Table Distribution of genotype in studies from meta-analysis. (DOCX)

S4 Table Distribution of genotype in studies from systematic review. (DOCX)

S1 File Meta-analysis on genetic association studies checklist. (DOCX)

S2 File PRISMA checklist. (DOC)
