# Supplementary material for: Association between matrix metalloproteinases polymorphisms and ovarian cancer risk: A meta-analysis and systematic review
Source: PLoS One. 2017 Sep 28;12(9):e0185456. doi: 10.1371/journal.pone.0185456 (PMC5619784; doi:10.1371/journal.pone.0185456)
Supplement: S2 Table — (DOCX) [file pone.0185456.s002.docx]

| gene | polymorphisms | homozygote model | heterozygote model | dominant model | recessive model | additive model |
| --- | --- | --- | --- | --- | --- | --- |
| MMP1 | rs1799750 | 1G1G vs 2G2G | 1G2G vs 2G2G | 1G1G+1G2G vs 2G2G | 1G1G vs 1G2G+2G2G | 1G vs 2G |
| MMP3 | rs34093618 | 5A5A vs 6A6A | 5A6A vs 6A6A | 5A5A+5A6A vs 6A6A | 5A5A vs 5A6A+6A6A | 5A vs 6A |
| MMP2 | C-1306T | CC vs TT | CT vs TT | CC+CT vs TT | CC vs CT+TT | C vs T |
| MMP2 | C-735T | CC vs TT | CT vs TT | CC+CT vs TT | CC vs CT+TT | C vs T |
| MMP7 | A-181G | AA vs GG | AG vs GG | AA+AG vs GG | AA vs AG+GG | A vs G |
| MMP8 | rs2155052 | CC vs GG | CG vs GG | CC+CG vs GG | CC vs CG+GG | C vs G |
| MMP8 | rs11225395 | CC vs TT | CT vs TT | CC+CT vs TT | CC vs CT+TT | C vs T |
| MMP9 | C-1562T | CC vs TT | CT vs TT | CC+CT vs TT | CC vs CT+TT | C vs T |
| MMP9 | rs6094237 | TT vs AA | TA vs AA | TT+TA vs AA | TT vs TA + AA | T vs A |
| MMP12 | rs2276109 | AA vs GG | AG vs GG | AA+AG vs GG | AA vs AG+GG | A vs G |
| MMP13 | rs17860523 | GG vs AA | GA vs AA | GG+GA=AA | GG vs GA+AA | G vs A |
| MMP20 | rs2292730 | GG vs AA | GA vs AA | GG+GA=AA | GG vs GA+AA | G vs A |
| MMP20 | rs12278250 | TT vs AA | TA vs AA | TT+TA vs AA | TT vs TA + AA | T vs A |
| MMP20 | rs9787933 | GG vs CC | GC vs CC | GG+GC=CC | GG vs GC+CC | G vs C |
