# Supplementary material for: Association between matrix metalloproteinases polymorphisms and ovarian cancer risk: A meta-analysis and systematic review
Source: PLoS One. 2017 Sep 28;12(9):e0185456. doi: 10.1371/journal.pone.0185456 (PMC5619784; doi:10.1371/journal.pone.0185456)
Supplement: S3 Table — (DOCX) [file pone.0185456.s003.docx]

| first author | gene | polymorphisms | case | | | control | | |
| --- | --- | --- | --- | --- | --- | --- | --- | --- |
|  |  |  | 1G1G | 2G1G | 2G2G | 1G1G | 2G1G | 2G2G |
| Kanamori [11] | MMP1 | rs1799750 | 18 | 84 | 61 | 30 | 56 | 64 |
| Biondi [12] | MMP1 | rs1799750 | 6 | 17 | 2 | 42 | 86 | 36 |
| Wenham [14] | MMP1 | rs1799750 | 86 | 147 | 78 | 101 | 204 | 82 |
| Li [18] | MMP1 | rs1799750 | 20 | 38 | 64 | 25 | 50 | 76 |
| Ju [19] | MMP1 | rs1799750 | 15 | 64 | 54 | 33 | 154 | 145 |
|  |  |  | 5A5A | 5A6A | 6A6A | 5A5A | 5A6A | 6A6A |
| Biondi [12] | MMP3 | rs34093618 | 3 | 19 | 3 | 42 | 74 | 48 |
| Smolarz [15] | MMP3 | rs34093618 | 37 | 46 | 35 | 26 | 52 | 32 |
| Li [18] | MMP3 | rs34093618 | 4 | 34 | 84 | 4 | 53 | 94 |
