# Supplementary material for: Association between matrix metalloproteinases polymorphisms and ovarian cancer risk: A meta-analysis and systematic review
Source: PLoS One. 2017 Sep 28;12(9):e0185456. doi: 10.1371/journal.pone.0185456 (PMC5619784; doi:10.1371/journal.pone.0185456)
Supplement: S4 Table — (DOCX) [file pone.0185456.s004.docx]

| first author | gene | polymorphisms | case | | | control | | |
| --- | --- | --- | --- | --- | --- | --- | --- | --- |
|  |  |  | CC | CT | TT | CC | CT | TT |
| Li [20] | MMP2 | C-1306T | 189 | 56 | 1 | 245 | 74 | 5 |
|  |  |  | CC | CT | TT | CC | CT | TT |
| Li [20] | MMP2 | C-735T | 164 | 69 | 13 | 181 | 127 | 16 |
|  |  |  | AA | AG | GG | AA | AG | GG |
| Li [18] | MMP7 | A-181G | 114 | 24 | 0 | 151 | 9 | 0 |
|  |  |  | CC | CG | GG | CC | CG | GG |
| Arechavaleta-Velasco [23] | MMP8 | rs2155052 | 33 | 2 | 0 | 34 | 3 | 0 |
|  |  |  | CC | CT | TT | CC | CT | TT |
| Arechavaleta-Velasco [23] | MMP8 | rs11225395 | 6 | 16 | 13 | 6 | 26 | 5 |
|  |  |  | CC | CT | TT | CC | CT | TT |
| Li [18] | MMP9 | C-1562T | 110 | 26 | 2 | 134 | 26 | 0 |
|  |  |  | TT | TA | AA | TT | TA | AA |
| Wang [24] | MMP9 | rs6094237 | 135 | 160 | 44 | 118 | 154 | 77 |
|  |  |  | AA | AG | GG | AA | AG | GG |
| Jia [22] | MMP12 | rs2276109 | 271 | 29 | 0 | 289 | 11 | 0 |
|  |  |  | GG | AG | AA | GG | AG | AA |
| Jia [22] | MMP13 | rs17860523 | 71 | 154 | 75 | 90 | 149 | 61 |
|  |  |  | GG | GA | AA | GG | GA | AA |
| Wang [24] | MMP20 | rs2292730 | 91 | 156 | 92 | 100 | 195 | 54 |
|  |  |  | TT | TA | AA | TT | TA | AA |
| Wang [24] | MMP20 | rs12278250 | 302 | 33 | 4 | 279 | 66 | 3 |
|  |  |  | GG | GC | CC | GG | GC | CC |
| Wang [24] | MMP20 | rs9787933 | 294 | 41 | 3 | 269 | 76 | 4 |
